# Synthesis and Characterization of Metakaolin–Wollastonite Geopolymer Foams for Removal of Heavy Metal Ions from Water

**DOI:** 10.3390/ma18030678

**Published:** 2025-02-04

**Authors:** Mazen Alshaaer, Bader Alharbi, Obaid Alqahtani, Mohammed S. Alotaibi, Abdullah Alzayed, Juma’a Al-Kafawein

**Affiliations:** 1 Department of Physics, College of Science and Humanities in Al-Kharj, Prince Sattam Bin Abdulaziz University, Al-Kharj 11942, Saudi Arabia; ba.alharbi@psau.edu.sa (B.A.); om.alqahtani@psau.edu.sa (O.A.); m.alotiaby@psau.edu.sa (M.S.A.); a.alzayed@psau.edu.sa (A.A.); 2 Department Mechanics of Materials and Constructions, Vrije Universiteit Brussels (VUB), Pleinlaan 2, 1050 Brussels, Belgium; 3 Department of Chemistry, King Faisal University, Al-Hassa 31982, Saudi Arabia; jalkafawein@kfu.edu.sa

**Keywords:** wollastonite, geopolymers, metakaolin, XRD, foam

## Abstract

Over the past few decades, researchers have focused on developing new compositions and preparation techniques for geopolymers, as multifunctional products, to optimize their characteristics for use in multiple applications. Therefore, this paper investigates metakaolin geopolymer foam and introduces new geopolymer foams based on hybrid metakaolin and wollastonite mineral precursors for water purification. The geopolymer foams were prepared using an alkaline activator, mineral-based powders (wollastonite and metakaolin), a foaming agent (aluminum powder), and a foam stabilizer (olive oil). In addition to mechanical tests and assessments of the adsorption capacity of heavy metal ions, the geopolymer foams were characterized using X-ray diffraction (XRD), scanning electron microscopy (SEM), and X-ray photoelectron spectroscopy (XPS). The geopolymer foams exhibited unique pore structures, containing four classes of pore networks with diameters around 1000 µm, 25 µm, 3 µm, and a well-arranged mesopore network of 50 nm. The utilization of wollastonite (CaSiO_3_) alongside metakaolin as a hybrid precursor led to fundamental changes in the composition of the geopolymer binders: a new crystal phase, Ca_5_(SiO_4_)_2_(OH)_2_, was formed, and the Si-Al-Na crystal phase disappeared, which led to an increase in the amorphous phase from 87% to 92%. The adsorption rate of heavy metal ions, namely Cr, Co, Cu, Zn, Pd, and As, increased upon introducing wollastonite as a precursor, with absorption rates ranging from 11% to 68%. The findings also revealed that wollastonite significantly increased the geopolymers foams’ compressive strength and elastic modulus from 30 KPa to 67 KPa and from 31 MPa to 126 MPa, respectively.

## 1. Introduction

Aluminum silicate-based powders are activated with alkali to form stable and hard materials with a tectosilicate structure through the process of geopolymerization [1,2,3,4,5]. The attractive features of geopolymers include their hardening at low temperature, their functional properties in various applications, and their excellent mechanical performance. They have a wide range of applications, from construction, waste recycling [6], and water treatment [7] to the stabilization of hazardous materials [8] and passive cooling systems [9]. However, using excessive amounts of alkaline solutions in field applications can pose a technical challenge. Additionally, the higher price of geopolymers compared to ordinary cement makes the large-scale use of this material difficult. To address these challenges, researchers are developing methods to promote geopolymers as green and environmentally friendly materials that can be used in engineering and environmental applications [10,11].

By properly heating kaolinite clays, metakaolin (MK), which has an amorphous structure, can be produced [12]. The addition of other fillers and precursors such as MK to geopolymer systems results in important improvements in the mechanical performance of the end geopolymer products, helping maintain good performance and minimize production costs [13]. Mesoporous and macroporous geopolymer-based materials undergo synthesis to become functional geopolymers. These materials serve the dual functions of improving acoustic and thermal insulation [14]. Zeolite is classified as an aluminosilicate-based mineral or tectosilicate that contains either alkali metals or alkali metal minerals. The properties of these minerals include adsorption, high ion exchange, heat resistance, and catalysis [13]. The alkali activation of zeolitic tuff and natural zeolites produces a geopolymer with excellent mechanical characteristics and a porous structure comparable to zeolites [15]. The primary constituents of laterite minerals are aluminum, iron, and aluminosilicates. Due to its corrosion resistance, laterite has traditionally been used as a building material and in roads and brick, distinctive for its typically reddish-brown color. Geopolymer production has become common due to laterite’s high mechanical performance. Due to its chemical composition, laterite is utilized as a raw material for Na-poly (sialate-siloxo) geopolymers because it has a better oxidation rate than other minerals [16]. Moreover, the molar oxide ratio of silica to alumina has a significant impact on the mechanical properties and microstructure of the laterite-based geopolymer. In addition, the process of blending laterite and other solid wastes creates high-strength geopolymers. The use of laterite and mixed laterite–slag geopolymers can be advantageous for non-load-bearing construction materials [17]. Geopolymers can be produced by using common natural minerals like bauxite [18], bentonite [19], diatomite [20], mullite [21], and halloysite [22], which are all aluminosilicate materials [13]. The amorphous features of certain natural minerals may require thermal treatment to increase their reactivity during geopolymerization reactions. The development of geopolymer materials via techniques with low energy consumption is a necessary direction for future research. Moreover, the mechanical performance of MK geopolymers can be improved when sulfur, a waste byproduct, is added to geopolymer precursors [17]. In addition, geopolymers with high mechanical performance can be produced using minerals such as wollastonite [23].

In recent years, there has been a sharp increase in interest in geopolymer foams (lightweight porous solid materials) [24,25,26]. The development of waste-based geopolymers, which are low-cost and environmentally friendly adsorbents, may be a motivating the approach to decontaminate industrial wastewaters and promote cleaner production processes [27,28]. The use of geopolymers to exchange cations with waste solution is a promising alternative to activated carbons, which has been recognized for a long time [29]. Although there are recent reviews available on the subject [25,30], they do not distinguish between the use of bulk-type and powdered geopolymer adsorbents. Al-Zboon et al. [31] found that geopolymer powders made of coal fly ash had an 81 mg/g lead uptake, while Cheng et al. found that powders made of metakaolin had a lead uptake nearly 100 mg/g [32]. Bai et al. [25] prepared a geopolymer block that was both floatable and permeable and applied the same concept to extracting dyes from wastewaters. The adsorption process involved the geopolymers being ground and passed through a 100 mesh sieve before they reached the surface. The powdered geopolymers were not easily retrieved or used directly in packed beds, even though they were reported to remove methylene blue at a very high rate (50.7 mg/g). Recently, the possibility of using monolithic bodies (e.g., membranes) instead of powders has attracted the interest of the scientific community, this being a safer and easier strategy in comparison with the use of nano- or micro-sized powders, allowing their direct use in packed beds without the need for supporting materials. A geopolymer cylindrical membrane based on metakaolin was employed by Ge et al. to extract Ni^2+^ from synthetic wastewater without requiring a post-separation step [33]. Novais et al. employed cylindrical discs in their extraction of lead from wastewater [34]. Despite the poor lead uptake shown by the geopolymer monoliths in comparison with powdered geopolymers, ranging from 0.95 to 6.34 mg/g [31], this was one of the first investigations concerning the use of monolith adsorbents (i.e., bulk-type absorbents as opposed to powders). The application of another bulk geopolymer adsorbent was documented in [35], where the maximum adsorption capacity for the geopolymer-supported zeolites was 37.9 mg/g. The authors of [36] report a cylindrical mesoporous adsorbent that can remove dye. The adsorbent’s SSA was 56.6 m^2^/g, and its average pore diameter was 20 nm. Basic violet and malachite green oxalate had maximum absorption capacities of 46.6 and 46.4 mg/g, respectively. The use of monolithic foamed geopolymers by Bai et al. [25] resulted in the extraction of copper and ammonium, with an 87% copper removal rate and a 95% ammonium removal rate, with an uptake of 0.54 and 0.57 mg/g, respectively. Novais et al. [26] reported another exciting approach, which is the use of metakaolin geopolymer spheres with a significant SSA (54 m^2^/g) and 60% total porosity. It was demonstrated that the developed spheres have an affinity for Cu^2+^, Pb^2+^, and Ca^2+^, and their maximum uptake values are 35, 46, and 24 mg/g, respectively. It was reported [37] that the removal capacity of elastomeric polymer nanocomposite foams for the Pb^2+^ ions exceeds 98%, and the removal of Hg^2+^ ions approaches almost 100% in the studied concentrations region of 20–40 ppm. Graphene-based aerogels/xerogels/cryogels (GBAs) have emerged and drawn significant attention as excellent materials for removing and recovering harmful and valuable metals from different aqueous media [38]. 

Recently, moving water purification units (MUGs) have been developed by our research group at Prince Sattam bin Abdulaziz University and registered as the patent under number SA 14328 [39]. These water purification units consist of geopolymer-based foam. One of the unique features of these water purification units is their use as moving materials, with saturation bulk densities lower than 1 g/cm^3^. Therefore, these geopolymer foam-based materials can be circulated by water currents on the surface of water bodies such as ponds, rivers, lakes, seas, reservoirs, etc. This study aimed to develop a geopolymer-based foam with acceptable mechanical performance, low saturation bulk density (less than 1 g/cm^3^), and high adsorption capacity of micropollutants. The microscopic, chemical, microstructural, and mechanical properties of these geopolymer foams are discussed and analyzed in this paper.

## 2. Materials and Methods

### 2.1. Materials

Kaolin clay, wollastonite, a Na_2_SiO_3_ solution, and NaOH were used during the process of synthesizing geopolymer cement. A kaolitic soil sample [23] was extracted from a deposit of kaolinite in Riyadh, Saudi Arabia, with the assistance of the Saudi Ceramic Company. The chemical composition of calcined kaolinitic soil (metakaolin) can be found in [23]. The precursor was estimated to contain approximately 90% kaolinite according to the loss of ignition. Kaolinite soil was heated in a Nabertherm (Lilienthal, Germany) furnace at 750 °C for 4 h to produce amorphous kaolinite (metakaolin). The secondary precursor used was wollastonite (CaSiO_3_) with fibrous crystals (NYAD^®^200), obtained from NYCO, Paris, France. Aluminum metal fine powder (purity 99.5%, ASSEEL Trading Corporation, Riyadh, Saudi Arabia) and organic olive oil (Aljouf, Saudi Arabia) were used as a foaming agent and stabilizer, respectively.

**Table 1:** Chemical composition of calcined kaolinite (metakaolin).

Compound	Composition%
MnO	0.34
Cr_2_O_3_	0.45
CaO	1.11
K_2_O	0.12
P_2_O_5_	0.93
Fe_2_O_3_	9.37
Al_2_O_3_	22.56
SiO_2_	38.41
TiO_2_	14.22

Na_2_SiO_3_, NaOH, and H_2_O were used to prepare the alkaline activator. Deionized water and pure pellets of sodium hydroxide (Merck, Darmstadt, Germany) were used to prepare the sodium hydroxide solution (NaOH). The Na_2_SiO_3_ solution consisted of 27 wt. % SiO_2_ and 8 wt. % Na_2_O [40].

### 2.2. Synthesis of the Geopolymer Foam

The molar ratio of Si/Al/Na was set at 1.6/1/1 for the preparation of solutions of sodium silicate, sodium hydroxide, and metakaolin powder. A 6.3:1 molar ratio of H_2_O/Na_2_O was present in the final alkaline solution. To prepare this alkaline solution, NaOH, H_2_O, and Na_2_SiO_3_ were stirred for 6 h. The powder component (metakaolin and wollastonite mixture) was added to the alkali solution and mechanically mixed. Olive oil (a stabilizing agent) and aluminum powder (foaming agent) were added after 10 min of mixing. Afterward, the components were again mixed for a period of 30 s. The geopolymer mixture was poured into silicone molds that had dimensions of 20 mm × 20 mm × 120 mm. After 60 min at room temperature, the molds were sealed and placed in an oven (Raypa Company, Barcelona, Spain) for curing at 40 °C for 24 h. After demolding, the resultant geopolymer foams were washed with distilled water for 6 h to remove any residual salts. The specimens were then subjected to various characterization methods.

**Figure 1:**
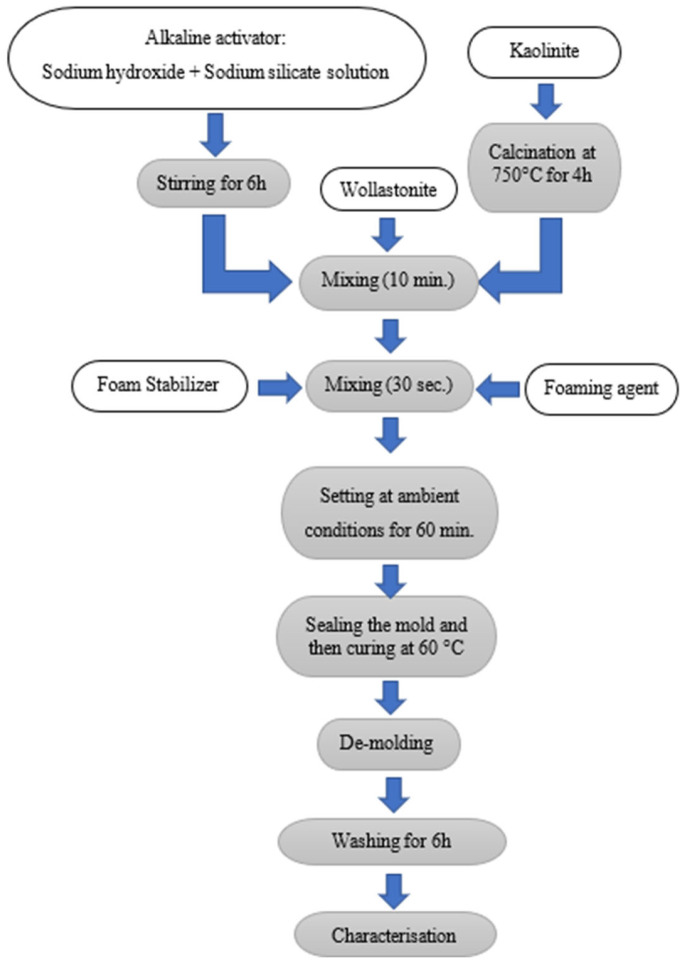
Experimental procedure of geopolymer foam sample preparation.

Figure 1 and Table 2 display the flowchart of the geopolymer foam preparation and the composition of the used ingredients, respectively. The sources of SiO_2_ include the alkaline solution and metakaolin. The source of sodium oxide (Na_2_O) was the alkali activator solutions, and aluminum oxide (Al_2_O_3_) was supplied by the metakaolin. The proposed precursor in this study, wollastonite, has the potential to release both CaO and SiO_2_ during its geopolymerization.

### 2.3. Characterization Techniques

The samples underwent phase analysis using a Rigaku Ultima IV XRD diffractometer-6000 (Rigaku Corporation, Tokyo, Japan) with a 2-theta scanning range of 10–60° at a 2°/min scan rate. MATCH! software was used to carry out a Rietveld refinement of the materials produced (Version 4, Crystal Impact, Bonn, Germany). The morphology and microstructure of the produced specimens were analyzed by coating the samples with gold and then performing SEM imaging (Quanta Inspect F50, FEI Company, Eindhoven, The Netherlands). The surface chemistry and the binding energy of the various powders were measured and recorded using elemental XPS (X-ray photoelectron spectroscopy) (Thermo K-Alpha spectrometer, Waltham, MA, USA).

A universal testing machine (HD-B615-S, Haida International Equipment Co., Ltd, Dongguan, China) was used to test, at room temperature, the compressive strength of the foams. This testing was performed on three specimens from each series. The dimensions of the specimens were 40 mm× 40 mm × 40 mm. The machine head was running at a speed of 2 mm per minute while being tested.

The water absorption (W_w_) of the samples was determined according to ASTM D570-98 [41]. Briefly, 2 mm plates were cut, always in triplicate. The samples were completely immersed in distilled water at room temperature (25 °C). The weight of each sample was then monitored until a constant weight was reached. The samples were weighed dry at the beginning and wet after soaking. The percentage of water absorption (W_w_) was then calculated using the following equation:

W_w_ = (W_f_ − W_i_)/W_i_ × 100

where W_i_ is the initial weight and W_f_ is the weight after absorbed water.

To assess the adsorption potential of the produced geopolymers foams, ~0.8 g samples of several geopolymeric cylindrical foams from each composition were immersed in standard solutions (100 ppm/L) of the following ions at pH = 5. pH (adjusted using NaOH/HClO_4_): Cr, Co, Cu, Zn, Pd, and As. After 12 h of shaking at 200 rpm, 10.0 mL samples from each solution were taken, filtered through microfilters (0.45 μm Nylon), and centrifuged prior to the determination of ion concentration using inductively coupled plasma optical emission spectrometry (ICP-OES) (THERMOSCI-ENTIFIC (iCAP 7000 series)) (Thermo Fisher Scientific, Waltham, MA, USA).

## 3. Results and Discussion

### 3.1. Microscopic Description

**Figure 2:**
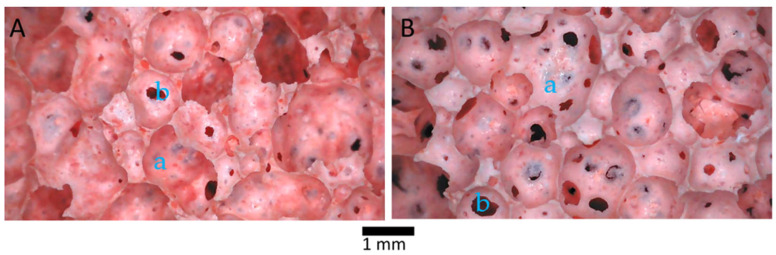
Images of geopolymer foam taken from optical microscope: (**A**) GKf and (**B**) GWf; (a) open cell and (b) closed cell.

Adding aluminum powder to geopolymer pastes resulted in the production of highly porous geopolymer foams with homogeneous pore size distributions. According to Figure 2, the diameter of the largest pore class is around 1 mm. Two types of structures are present in the cellular structures of both GKf and GWf: an open cell system and a closed cell system. Due to their dual open and closed cell structure, these geopolymers offer a distinctive combination of excellent structural performance and superior functional characteristics, including being lightweight and offering high water absorption [26]. The effectiveness of water purification is enhanced by the increased absorption capacity for water and solutions facilitated by the open cells. Meanwhile, the closed cells play a significant role in keeping the saturated bulk density below 1 g/cm^3^ (water density) for MUG synthesis (Table 3) [39].

Table 3 shows that GKf and GWf have bulk densities of 0.20 and 0.21, respectively. The bulk densities after saturation for GKf and GWf are 0.54 g/cm^3^ and 0.74 g/cm^3^, respectively, which correspond to the required characteristics of developing floatable geopolymer units. The proportion of open cells increases when wollastonite is added, as reported in Table 3. Water absorption, which is proportional to the distribution of the open pore system, is 156% and 276% for GKf and GWf, respectively. The water purification capacity of geopolymer foams is enhanced by their high-water absorption, especially GWf’s.

**Figure 3:**
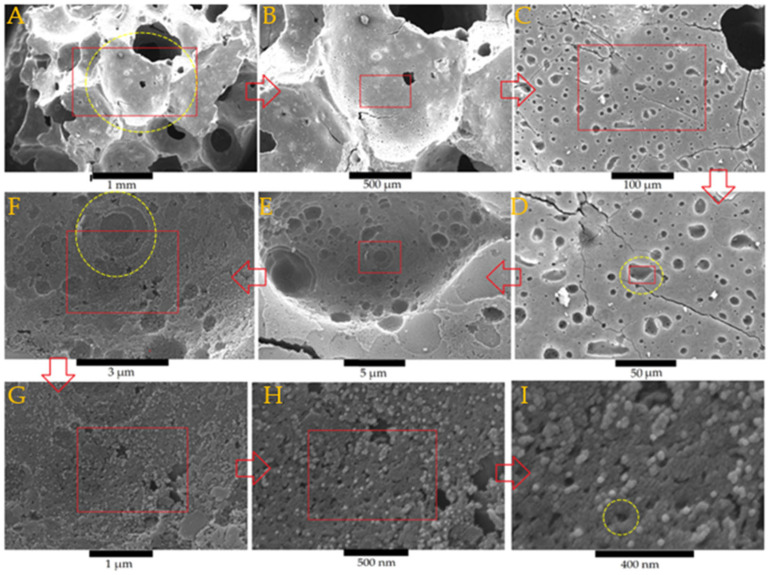
SEM images of a geopolymer foam, GKf, with different magnifications. Rectangle: the region subjected to further magnification; circle: pore class.

**Figure 4:**
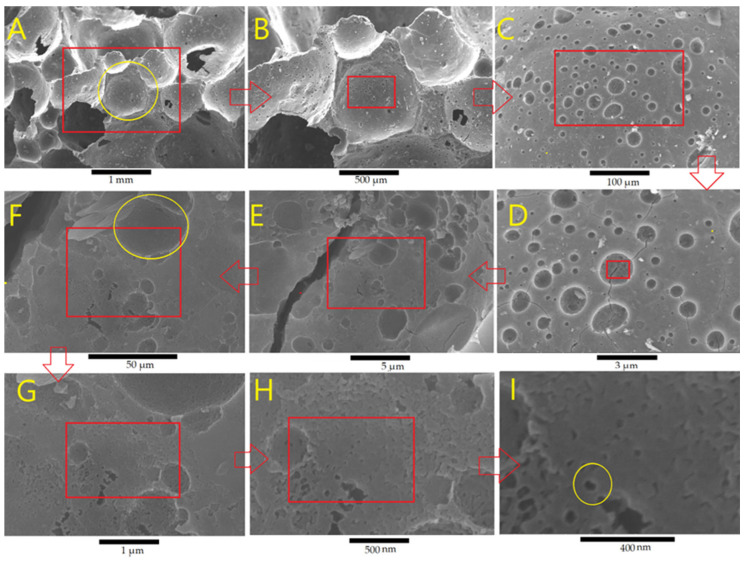
SEM images of a geopolymer foam, GWf, with different magnifications. Rectangle: the region subjected to further magnification; circle: pore class.

Figure 3A–I show SEM images of the geopolymer foam, GKf, at different magnifications. GKf is characterized by its primary spherical macropore network, first pore class (diameter around 1 mm), micro pore wall, and micro pore strut (Figure 3A,B). Further magnification (Figure 3B,C) shows that the wall of the first pore class contains scattered fine pores of the second pore class, with a size around 25 μm (Figure 3D,E). The walls of the second class of pores (Figure 3F), which constitute the third pore class, show smaller pores measuring 1 to 3 µm. Finally, a mesopore network of around 50 nm was observed in the third pore network class, as reported in Figure 3, and the fourth pore class [10]. Similar pore structures and classes can be observed in GWf, Figure 4A–I, except for some differences in the pore morphology where more regular shapes can be observed in GWF (Figure 4C,D).

The results above show that the foam has a unique pore system that overlaps four different classes of pores to form a hierarchical structure with approximate diameter sizes of 1000, 25, 3, and 0.05 µm.

### 3.2. Analysis of Microstructure and Phase Composition

To determine the role of foaming in the matrix of geopolymers, an XRD analysis was carried out. Table 4 shows XRD scan analyses of the MK geopolymer (GK), MK geopolymer with foam (GKf), the hybrid wollastonite–MK geopolymer (GW), and the wollastonite–MK geopolymer with foam (GWf). XRD analyses of kaolinite and wollastonite as precursors are presented in our previous study [23].

As a result of geopolymerization, a high background for the XRD patterns, between 15° and 35°, can be seen in Table 4, confirming the presence of an amorphous phase in the produced MK geopolymer (GK) [40]. According to the Rietveld refinement analysis of the XRD patterns, the degree of GK crystallinity was 42% and crystalline phases were detected: monoclinic and triclinic AlNaO_8_Si_3_ (Albite) and Na_6_O_19_Si_8_ (Table 4). The foaming effect slightly impacts the phase composition of the MK geopolymer, where the degree of crystallinity is reduced from 42% (GK) to only 13% (GKf). The same phases are observed in both the MK geopolymer (GK) and its foam variant (GKf).

As shown in Figure 4, introducing wollastonite to the MK geopolymer diminished all the peaks corresponding to AlNaO_8_Si_3_ (Albite) and Na_6_O_19_Si_8_ and formed new crystalline phases (Ca_5_(SiO_4_)_2_(OH)_2_) and calciochondrodite minerals, in addition to several peaks corresponding to wollastonite. The foaming GW (GWf) geopolymer showed a degree of crystallinity from 20% to 8%, without changes in the phase composition, as reported in Table 4. In addition to the increase in the amorphous phase in both geopolymers, GK and GW, as a result of foaming, there is a significant reduction in the crystallite size. Both crystalline phases of GK, Na_6_O_19_Si_8,_ and AlNaO_8_Si_3_, reduced from 1512 Å and 5484 Å to 715 Å and 174 Å, respectively. Also, the crystallite sizes of the GW phases, Ca_5_(SiO_4_)_2_(OH)_2_ and CaSiO_3_, were reduced from 2691 Å and 3699 Å to 486 Å and 962 Å, respectively.

The observable reduction in the unit cell of wollastonite (CaSiO_3_) from 794.3 Å (GW) to 398 Å (GWf) after foaming is an indication that this phase was distorted during the foaming process. There was also a decrement in wollastonite unit cells from 794.3 Å^3^ to 398 Å^3^ after foaming (Table 4), which could be a result of recrystallization, a secondary phase, or the partial dissolution of wollastonite during foaming.

**Figure 5:**
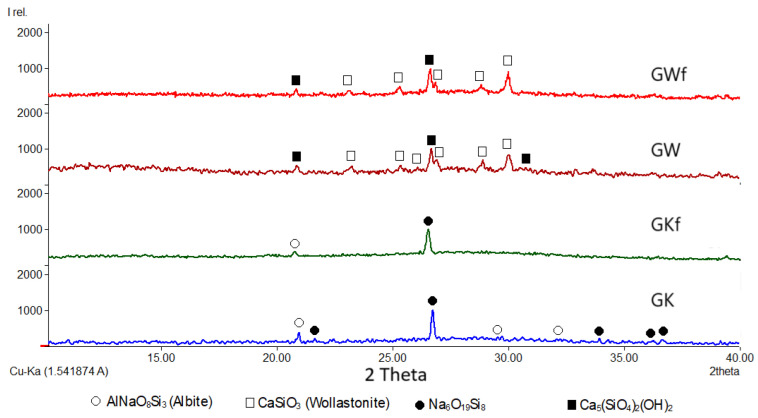
Qualitative XRD patterns for metakaolin geopolymer (GK), wollastonite–metakaolin geopolymer (GW), and corresponding GKf and GWf foams.

Figure 5 depicts GKf’s microstructure, composed of open pores (point a), the geopolymer binder (point b), and partially dissolved metakaolin (point c). The EDS analysis of area (1) shows that the geopolymer binder is composed of a Na-Al-Si matrix with a molar ratio of 1:1.3:1.8. These molar fractions are comparable with those reported in previous studies [42]. The other detected phase comprises leftover metakaolin layers as the primary precursor (point c). The EDS analysis of area 2 shows that the Si/Al molar ratio is around 1, which is similar to that of metakaolin. On the other hand, the presence of Na and C ions indicates the formation of Na_2_CO_3_ as a result of the carbonation of the residual alkaline activator.

**Figure 6:**
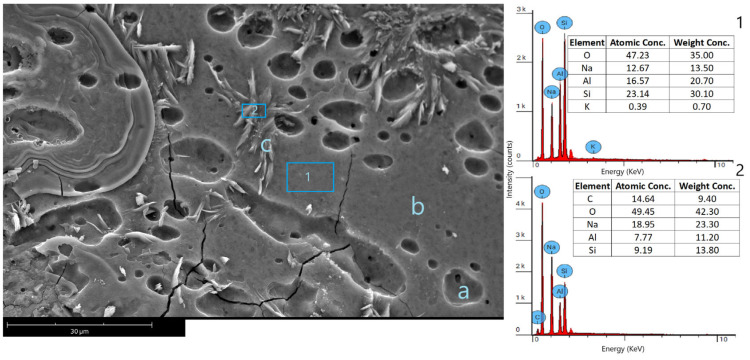
SEM image and EDS analysis of MK geopolymer foam (GKf); (a) pores, (b) geopolymer binder, and (c) metakaolin layers.

The microstructure of GWf is composed of different combinations of Na-Al-Si and Ca-Si geopolymer binders, as determined by the EDS analysis (Figure 6). Amorphous silica is observed in area 4, where SiO_2_ is formed (Figure 6), without the corresponding XRD peaks (Figure 4). This is an indication that the incorporation of wollastonite (CaSiO_3_) during geopolymerization results in the release of Ca ions, causing the amorphous structure of SiO_2_. A summary of the compositions of areas 1, 2, and 3 is given in Table 5. It is observed that the deviation in the Si/Al/Na molar ratios from one area to another is relatively small, while the deviation in Ca is quite large. This is a result of the high contribution of metakaolin as the sole source of Al compared with the partial dissolution of wollastonite (Figure 4) as a source of Ca, which is already present in a smaller amount (Table 2).

### 3.3. Mechanical Characterization of the Geopolymer Foams

**Figure 7:**
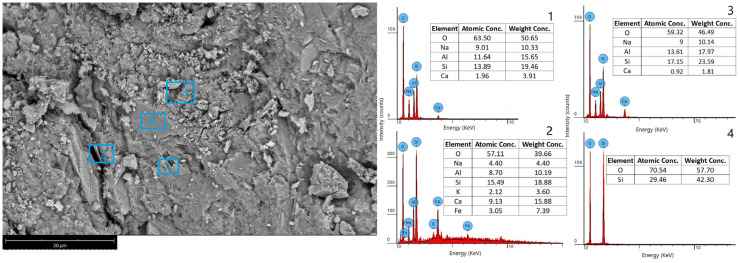
SEM image and EDS analysis of wollastonite–MK geopolymer foam (GWf).

Compressive strength, or crushing strength, is commonly used to measure the mechanical strength of brittle foam materials, such as geopolymer foams. The GKf and GWf specimens have a typical compressive stress–strain graph, as depicted in Figure 7. The stress–strain curve oscillates during testing due to the cell being crushed and collapsed layer by layer when stress is above the yield limit of the cell walls of the foamed geopolymer (Figure 7) [43]. The stress increases significantly with an increase in strain when all layers of cells are crushed into a lock [43]. Typical foam behavior is observed during compressive loading conditions for the compressed GKf and GWf geopolymer foams. These stress–strain curves are composed of three distinguishable zones: (1) a linear elastic zone, where the cell walls buckle elastically; (2) a plateau zone, where the cells begin to undergo plastic deformation and collapse, starting with the plateau stress (σ_p_); and (3) a densification zone, where the cells are completely collapsed and the stress rises significantly, the onset of which is characterized by the densification strain (ε_d_).

In the initial linear elastic region, it is observed that GWf has a higher yield strength of 67 Kpa compared to that of GKf, which is 30 KPa. The addition of wollastonite leads to an increase in the elastic modulus from 31 MPa (GWf) to 126 MPa (GWf). The cell size (Figure 2) and the bulk density of the geopolymer foams (Table 3) are similar; thus, the significant improvement in the mechanical performance of GWf is an indication of the improved nature of the geopolymer matrix by adding wollastonite. The cells begin to collapse beyond the elastic region and continue to collapse throughout the plateau region. It was noticed that the plateau region of the GWf specimens is longer than that of the GKf specimens. The cells in the remaining foam are filled with debris from cell collapse, which causes an increase in foam density and causes stress in the densification region [44]. In all regimes, GWf specimens experience the highest level of stress, resulting in their highest strength under compression.

### 3.4. Effect of Wollastonite on Adsorption Potential of Geopolymer Foam

**Figure 8:**
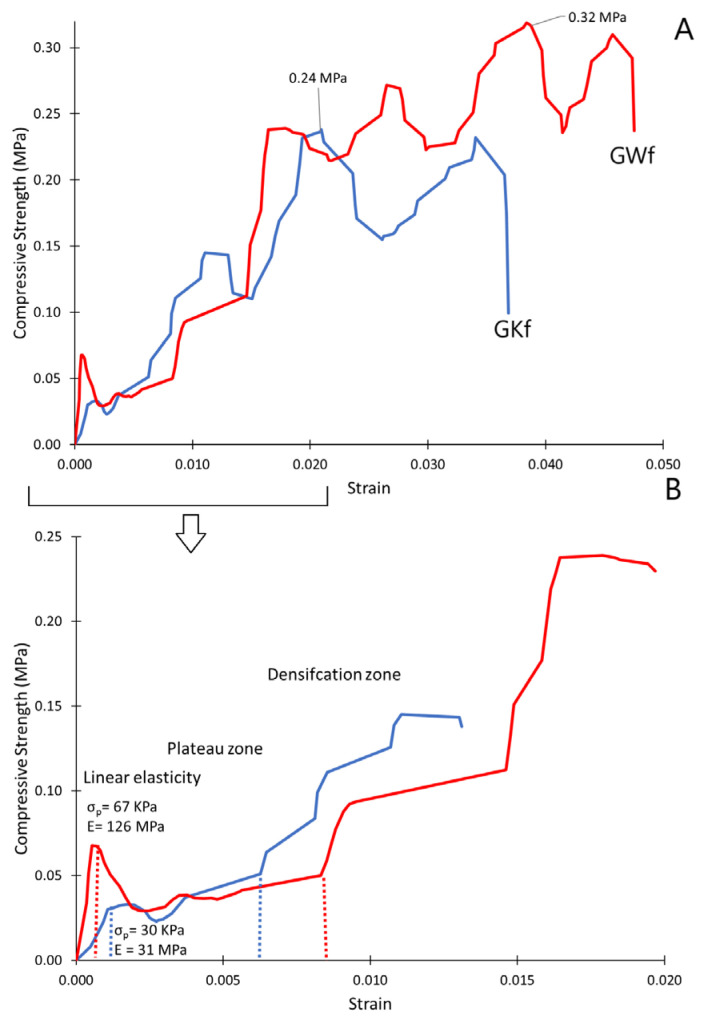
Compressive strength of geopolymer foams (GKf and GWf); (**A**) stress-strain curve, and (**B**) the initial stage of the stress-strain curve.

In order to evaluate the effect of wollastonite on the adsorption properties of the geopolymer foam, the adsorption of Cr, Co, Cu, Zn, Pd, and As ions onto the GKf and GWf geopolymers foams was investigated as a preliminary study. The aim of the adsorption investigation was to compare the adsorption capacities of the GKf and GWf specimens instead of assessing the adsorption mechanism, which has been studied elsewhere [1]. GWf shows a much higher adsorption rate of Cr, Co, Cu, Zn, Pd, and As ions than GKf, as seen in Figure 8. The adsorption rate of heavy metal ions, namely Cr, Co, Cu, Zn, Pd, and As, by the geopolymer foam increased by 33%, 31%, 62%, 68%, 31%, and 11%, respectively, by adding wollastonite as a precursor (GWf). Based on these findings, geopolymer foams can be utilized for the adsorption of heavy metal ions in acidic conditions and for the subsequent treatment of wastewater and industrial effluents.

By summarizing the experimental results in this study, we can conclude that the formation of a nanoporous mineral matrix during geopolymerization occurs, as indicated in the present study and shown in Figure 3. Foaming results in a higher surface area and thus greater adsorption potential. According to the XRD patterns of GWf, geopolymerization results in the disappearance of most wollastonite peaks (Figure 4). Geopolymerization leads to a decrease in crystallinity for geopolymer foam and a more amorphous structure, as depicted in Table 4, which leads to an increase in adsorption sites [1]. The process of absorbing heavy metal ions involves cation exchange with Na^+^ and Ca^+2^. This is plausible since the geopolymerization of the precursors involves the use of a very alkaline solution. The Na and Ca ions in the alkali solution play a significant role in neutralizing the electric potential of the geopolymer foam by substituting hydrogen ions present in the broken edges of the aluminosilicate layers. The amorphous Na-Si-Al-Ca matrix is subsequently formed.

### 3.5. The Elemental Composition of Geopolymer Foam Before and After Adsorption of Heavy Metal Ions

**Figure 9:**
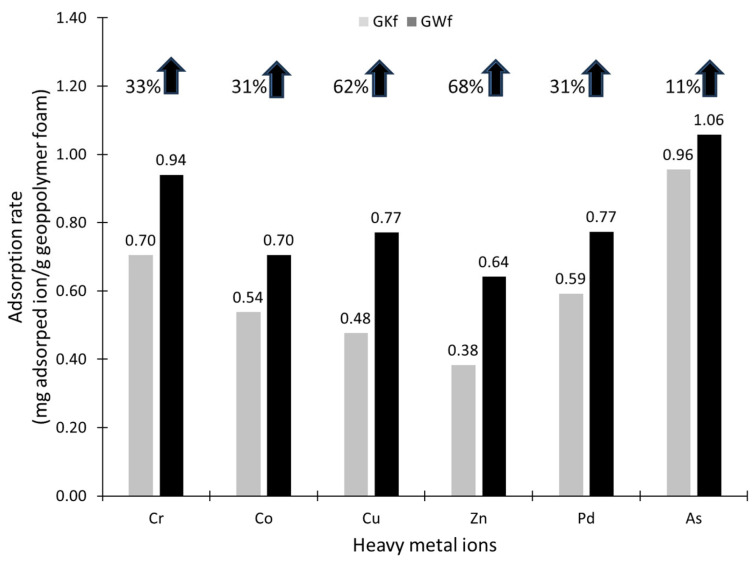
Adsorption of heavy metals ions by geopolymer foams (one solution for all the elements; ion concentration:, 100 ppm; 10 mL solution/0.8 g of geopolymer; duration, 6 h; pH = 5).

**Figure 10:**
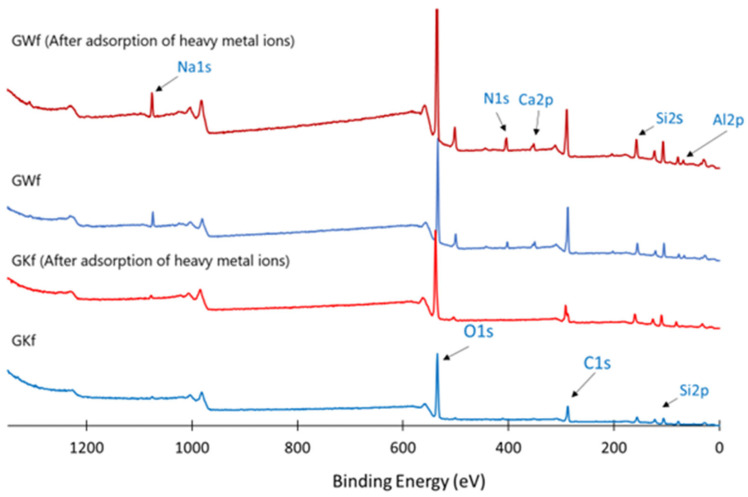
XPS spectra of geopolymer foams before and after adsorption of heavy metal ions (Figure 8).

XPS analysis is conducted to examine the chemical states of Na, Al, Si, and Ca in the GKf and GWf geopolymer foams before and after the adsorption of heavy metal ions (Figure 9), focusing on their surface chemistry, as depicted in Figure 10. Distinct peaks are identified in the XPS spectra of GKf that are connected to the Na 1s, Al 2p, and Si 2p orbitals. In addition to the peak of GKf, the XPS analysis of GWf reveals peaks that are associated with Ca 2p. In GWf, the intensity of the Na 1s peak is higher, which suggests that there is a higher rate of geopolymerization when wollastonite is added as a secondary precursor to the geopolymer mix.

Table 6 show that wollastonite has an impact on the binding energies of Al 2p, Si 2p, and Na 1s and their respective peaks. The binding energies of the Al 2p, Si 2p, Si 2s, and Na 1s peaks in GWf decreased slightly by a few hundred electrons between 77 and 76 eV, 104 and 105 eV, 154 and 159 eV, and 1075 eV and 1074 eV, respectively. As shown in Table 2, the existence of Ca in the geopolymer matrix, caused by the use of wollastonite as a precursor, results in the confirmed Ca 2s peak at 403 eV in GWf only. Table 6 shows that the binding energies of Al 2p, Si 2p, Si 2s, Na 1s, and Ca 2s increase, ranging from 1 eV to 5 eV, following water treatment with these foams. These changes in the binding energies of Al 2p, Si 2p, Na 1Ss, and Ca 2s after the adsorption of the heavy metal ions (Figure 8) are indications of the ion exchange taking place within these metal ions.

## 4. Conclusions

The main objective of this study was to synthesize and characterize geopolymer foam. The developed geopolymer foam has a unique pore structure that contains four classes of pore networks with diameters of around 1000 µm, 25 µm, and 3 µm and a final well-arranged mesopore network of 50 nm. The integration of these pore classes was confirmed via SEM analysis, with smaller networks branching out through the walls and struts of larger pores in a unique hierarchical pore system. It was also found that geopolymer foam contains both closed and open pores. Closed pores are important to maintain the bulk density of a foam, even at saturation levels lower than the water density, thus allowing it to float or move with the water current during purification. In addition, the open pore network improves water purification capacity by increasing water absorption. This study introduced a geopolymer foam that uses both metakaolin and wollastonite, called GWf, as a precursor and compared its properties with those of GWf, a geopolymer foam that is based on metakaolin alone.

This study shows that adding wollastonite as a precursor alongside metakaolin leads to a complete change in the geopolymer foam’s microstructure, phase composition, adsorption capacity, and mechanical characteristics. The disappearance of crystalline Na-Si-Al phases, namely Na_6_O_19_Si_8_ and AlNaO_8_Si_3_, is observed through X-ray analysis, and a new crystalline phase, calciochondrodite Ca_5_(SiO_4_)_2_(OH)_2_, emerges. This phase may be crucial in the development of strong C-S-H bonds. According to the study’s findings, wollastonite can have a significant impact on the microstructure of the geopolymer foam. As a result of incorporating wollastonite and metakaolin as precursors, the amorphous phase increases from 87% to 92%. When wollastonite is used as a precursor, heavy metal ions, such as Cr, Co, Cu, Zn, Pd, and As, have an increased adsorption rate, with ranges from 11% to 68%. The findings also reveal that wollastonite significantly increases the compressive strength and elastic modulus of the geopolymer foam from 30 KPa to 67 KPa and from 31 MPa to 126 MPa, respectively.

This preliminary study undoubtedly presents many opportunities and challenges for developing promising new hybrid geopolymer foams. The studied foams show attractive physical, mechanical, and adsorption characteristics for different applications, particularly in mobile water purification units for water treatment.

## Figures and Tables

**Table 2:** Mixes and compositions of the prepared geopolymer foams.

	Metakaolin (g)	Wollastonite (g)	Na_2_SiO_3_ (g)	NaOH (g)	H_2_O (g)	Olive Oil (ml)	Aluminum Powder (g)
GK	100	0	100	25	48	0	0
GW	100	25	100	25	48	0	0
GKf	100	0	100	25	48	1	0.4
GWf	100	25	100	25	48	1	0.4

GK: metakaolin-based geopolymer, GW: metakaolin–wollastonite geopolymer, GKf: metakaolin-based geopolymer foam, and GWf: metakaolin–wollastonite geopolymer foam.

**Table 3:** Physical properties of the geopolymer foams.

	ρ (g/cm^3^)	ρ_sat_ (g/cm^3^)	W_w_
GKf	0.21	0.54	156
GWf	0.20	0.74	276

ρ: bulk density (mass/bulk volume), ρ_sat_: saturated density (mass after water absorption/bulk volume), and W_w_: water absorption (weight of water absorbed by a material in saturated state over weight of dry material).

**Table 4:** Rietveld refinement of XRD data showing the phase composition, crystallinity, and crystal parameters of the geopolymers and their corresponding foams (MATCH! software, version 4).

	Degree of Crystallinity	Crystalline Phase Composition	Phase %	Crystal System	Unit Cell Size (Å^3^)	Crystallite Size (Å)
GK	42%	Na_6_O_19_Si_8_	55.6	monoclinic	1772	1512
		AlNaO_8_Si_3_	44.4	triclinic (anorthic)	626	5484
GKf	13%	Na_6_O_19_Si_8_	52.4	monoclinic	1772	715
		AlNaO_8_Si_3_	47.6	triclinic (anorthic)	666	174
GW	20%	Ca_5_(SiO_4_)_2_(OH)_2_	29.6	monoclinic	486.1	2691
		CaSiO_3_	70.4	monoclinic	794.3	3699
GWf	8%	Ca_5_(SiO_4_)_2_(OH)_2_	17.7	monoclinic	486.1	486
		CaSiO_3_	82.3	triclinic (anorthic)	398	962

**Table 5:** Molar ratios of Si, Al, Na, and Ca in the microstructure of GWf.

Region #	Si/Al	Na/Al	Si/Ca
1	1.2	0.8	7.1
2	1.8	0.5	1.7
3	1.3	0.7	18.6

**Table 6:** The XPS analysis of the chemical state of the Ca 2s, Na 1s, Si 2p, Si 2s, and Al 2p orbitals in the GKf and GWf geopolymer foams before and after the adsorption of heavy metal ions.

	Binding Energy (eV)				
	Ca 2s	Na 1s	Si 2p	Si 2s	Al 2p
GKf	-	1075	105	155	77
GKf (after adsorption)	-	1078	109	159	82
GWf	403.08	1074.08	104	155	76
GWf (after adsorption)	404.08	1076.08	106	157	78

## Data Availability

The data that support the findings of this study are available from the corresponding author, M. Alshaaer, upon reasonable request.
